# Urban Air Pollution and Emergency Department Visits for Neoplasms and Outcomes of Blood Forming and Metabolic Systems

**DOI:** 10.3390/ijerph19095603

**Published:** 2022-05-05

**Authors:** Mieczysław Szyszkowicz, Anna Lukina, Tatiana Dinu

**Affiliations:** 1 Environmental Health Science & Research Bureau, Health Canada, Ottawa, ON K1A 0K9, Canada; anna.lukina@hc-sc.gc.ca; 2 Water and Air Quality Bureau, Health Canada, Ottawa, ON K1A 0K9, Canada; tatiana.dinu@hc-sc.gc.ca

**Keywords:** abdominal and pelvic pain, ambient air pollution, benign neoplasm, diabetes mellitus, relative risk

## Abstract

This study focused on investigating possible associations between exposure to urban air pollution and the number of emergency department (ED) visits for various health outcomes. The outcomes were grouped into four chapters of the International Classification of Diseases Tenth Revision (ICD-10) system (i.e., Chapter II-IV: “Neoplasms”, “Diseases of the blood”, “Endocrine, nutritional and metabolic diseases”, and XVIII: “Symptoms, signs and abnormal clinical and laboratory findings“). The data were collected for the city of Toronto, Canada, (2004–2015, 4292 days). Four gaseous air pollutants (carbon monoxide (CO), nitrogen dioxide (NO_2_), ground level ozone (O_3_), and sulfur dioxide (SO_2_)) and fine particulate matter (PM_2.5_), and two calculated air quality health indexes (AQHI) based on Toronto were used. The statistical models were constructed by applying the conditional Poisson regression. The exposure was assessed over a maximum of 15 days (time lags 0–14 days). An analysis was performed with the following strata: sex, age, and seasons. Relative risks (RR) and their 95% confidence intervals (95%CI) were estimated for an increase in concentration by a one interquartile range (IQR). For the AQHI (composed of NO_2_, O_3_, and PM_2.5_), IQR = 1, the estimations for lag 1 and all patients, are RR = 1.023 (95%CI: 1.008, 1.038), 1.026 (1.012, 1.040), 1.013 (1.003, 1.024), and 1.007 (1.003, 1.010) for Chapters II–IV and XVIII, respectively. The results show that in the four large, analyzed health groups, the impact of air quality mainly occurs over a short period (from current day to a maximum of 3 days after exposure).

## 1. Introduction

There is growing evidence of the positive associations between exposure to ambient air quality and various health conditions starting from respiratory outcomes to various cardiac problems. For instance, short-term exposure to fine particulate matter of 2.5 μm or less in aerodynamic diameter (PM_2.5_) was positively associated with hospital admissions for seven major disease categories (e.g., endocrine, nervous system diseases, digestive diseases, nutritional and metabolic diseases, circulatory diseases, respiratory diseases, musculoskeletal and connective tissue diseases, and genitourinary diseases) [1,2,3]. Additionally, similar associations were observed for 35 minor disease categories and the same-day PM_2.5_ exposure in both single and two-pollutant models (e.g., chronic ulcer of skin, diabetes mellitus, anemia, liver diseases, intestinal infection, gastrointestinal hemorrhage, urinary tract calculus, renal failure, and back problems) [4].

Ambient daily exposure to PM_2.5_, particulate matter of 10 μm or less in aerodynamic diameter (PM_10_), sulphur dioxide (SO_2_), and ground-level ozone (O_3_) was positively associated with lung cancer mortality defined as code C33 (malignant neoplasms of the trachea) and code C34 (malignant neoplasms of bronchus and lungs) according to the International Classification of Diseases version 10 (ICD-10; WHO, 2016) [5,6]. Furthermore, the study showed that the associations varied by city and season, daily air pollutants, and weather conditions, and had synergistic effects on the daily lung cancer mortality. The authors noticed that older people and male lung cancer patients were the most susceptible to air pollution exposure. Short-term exposure to SO_2_ was associated with increased lung cancer mortality (ICD 10 codes: C33–C34) in males and varied by season [7].

Daily exposure to ambient PM_2.5_ was significantly associated with an increase in the prevalence of moderate and severe anemia (ICD-10 codes: C65 and C64, respectively), and decreased hemoglobin values in children under 5 years of age [8]. Short-term exposure to ambient fine particulate matters (PM_2.5_), carbon in atmospheric particulate matters, ultrafine particles, and accumulated mode particles was significantly associated with levels of anemia-related blood cell (ICD-10 codes: D50–D89) parameters levels in elderly, such as red blood cell count, hemoglobin, hematocrit, mean corpuscular hemoglobin, mean corpuscular hemoglobin concentration [9].

Exposure to volatile organic compounds (VOCs), carbon monoxide (CO), and nitrogen dioxide (NO_2_) was correlated to the odds of primary hypothyroidism (ICD-10 code: E03) in residents living near a petrochemical complex [10]. Short-term exposure to PM_2.5_ on lag 0 day was significantly associated with increases in hospital outpatient visits for endocrine (ICD-10 codes: E00–E90), digestive, urological (ICD-10 codes: R30–R39), and dermatological diseases [11]. Daily exposure to dust outbreaks containing high concentrations of PM_10_ and PM_2.5_ was significantly associated with respiratory emergency department visits (ICD-10 codes: R04.2–R06, R09.0–R09.3, R09.89) especially in the ≥65 year and ≤5 year age group [12].

In this study, it was hypothesized that exposure to urban air pollutants is positively associated with the adverse health outcomes grouped in the following four ICD-10 categories: (a) ICD-10 codes: C00–D48: Neoplasms (presented in Chapter II); (b) ICD-10 codes: D50–D98: Diseases of the blood and blood-forming organs and certain disorders involving the immune mechanism (presented in Chapter III); (c) ICD-10 codes: E00–E90: Endocrine, nutritional and metabolic diseases (presented in Chapter IV); and (d) ICD-10 codes: R00–R99: Symptoms, signs and abnormal clinical and laboratory findings, not elsewhere classified (presented in Chapter XVIII).

The primary purpose of the paper is to provide evidence that various health conditions, often not yet studied, are associated with air pollution. The objective of the present study is to show the associations of health problems in relation to air pollutants, their lags, and the considered subgroups (age, sex, and season). The research gap has mainly been created by the history of environmental epidemiology, where for a long period of time the respiratory conditions dominated such studies. Successively but slowly, other health problems were introduced and considered, by adding cardiac conditions, various aspects of mental health, and others. Probably, the domain that is still dominated by respiratory conditions, will differ markedly if the hypothesis that air pollution may be affecting every organ in the human body holds true. This presentation uses widely accepted statistical methodologies and reliable and well-established health and environmental data. The results will add to knowledge on the burden of disease in relation to acute changes in urban ambient air pollution concentrations.

## 2. Materials and Methods

The present study accounted for patients’ sex and age groups (0–60+ years) among those attending the emergency department (ED) of Toronto hospitals. In addition, the possible effects of seasons (“cold” expressed as October-March and “warm” expressed as April-September) during the study were also considered, which covered the overall period between April 2004 and December 2015 (total of 4292 days). The two seasons differ by the mean temperature, which in the cold season was 1.6 [minimum–maximum: −22.2–23.5] and in the warm season was 17.0 [−4.2–31.2]. The values are given in degrees Celsius.

### 2.1. Health Data

The boundaries of the study area are well defined by the Census Division (CD) of Toronto, one of the largest and most populous urban cities in Ontario. The considered region is approximately 630.2 square kilometers. Based on the 2016 CD, the population density of Toronto is 4334 people per square kilometer. The enumerated population of Toronto in the year 2016 was 2,731,571 persons. Daily ED visits by location within the well-determined CD of Toronto were linked using the alphanumeric postal codes. These six-character strings for each patient’s home address were used to include the patients in the study.

The National Ambulatory Care Reporting System (NACRS) database was used as a source of the health outcomes data in this study (NACRS, 2020; [13]), based on the Canadian Institute Health Information (CIHI) reporting system [14]. The NACRS system contains more than 97% of the ED visits in the province of Ontario, in which Toronto is the capital. The database used is a health reporting system and among other health records contains patients’ data on various primary diagnoses during the ED visits. The data were retrieved for the period between 1 April 2004 and 31 December 2015, inclusively, for a total of 4292 days. The daily ED visits were identified using the primary cause of emergency department visits registered in the analyzed health records. The cases were categorized using the International Classification of Diseases Tenth Revision (ICD-10) codes [5] (WHO, 2016). The following health conditions grouped in the four chapters of this classification system were analyzed: Chapter II: (C00–D48) labelled as “Neoplasms”, Chapter III: (D50–D89) labelled as “Diseases of the blood and blood-forming organs and certain disorders involving the immune mechanism”, Chapter IV: (E00–E90) labelled as “Endocrine, nutritional and metabolic diseases”, and lastly Chapter XVIII: (R00–R99) labelled as “Symptoms, signs and abnormal clinical and laboratory findings, not elsewhere classified”. The analysis was performed separately for each of the four health outcomes. To investigate any possible modifying effects of patients’ sex (male, female, and all), age (from 0 to 60+ years old), and influence of seasons (warm season defined as the period between April and September and cold season defined as the period between October and March) on the ambient air quality associations with the analyzed four health outcomes the 18 strata were defined. The strata were constructed using sex, age group, and season.

### 2.2. Environmental Data

Long-term ambient air pollution data and meteorological conditions data were collected by the National Air Pollution Surveillance (NAPS) monitoring program, maintained by Environment and Climate Change Canada (NAPS, 2020; [15]). The data were collected by daily averages of 24 h measurements using seven municipal fixed-site monitoring stations with an approximate maximum distance among them of 15 km. There were nine air pollution monitoring stations with two stations paired (very close to each other) and the twins operated exclusively and alternatively. The data were collected every hour (total of 24 h) and on a daily basis for the entire course of the study. Five major air pollutants were considered, such as carbon monoxide (CO), nitrogen dioxide (NO_2_), ground-level ozone (O_3_), sulfur dioxide (SO_2_) and fine particulate matter (PM_2.5_). Ground-level O_3_ was also measured at a maximum 8 h average since such a pollutant can reach its highest concentration during sunlight hours. On the other hand, meteorological data including daily ambient mean temperature and relative humidity were collected from one monitoring station only located at the Toronto airport [16]. The composite Air Quality Health Index (AQHI), which unites individual pollutants, such as NO_2_, O_3_ and PM_2.5_, concentrations measured in only Toronto was also calculated. The AQHI is expressed using an 11-point scale (from 1 to 10, and 10+), where lower points mean low risks to human health and higher points mean higher risks to human health. The derivation, interpretation and discussion of the methodological approach on calculating AQHI is discussed in detail in [17]. In a similar way, the AQHIX was defined by using O_3_-8h rather than O_3_ in the formula.

Exposure data were assessed at a maximum of 15 days (lags from 0 to 14 days) to consider any potential delayed effects of elevated air pollution levels that persisted for several days.

### 2.3. Statistical Methods

A time-stratified case-crossover design was applied to examine the associations between air pollutants and the considered adverse health outcomes grouped in the chapters [18,19]. This design has been widely and commonly executed to investigate the associations of short-term air pollution exposures on the risk of acute health outcomes. The technique compares exposure levels between the event time (‘case’) and the matched control periods (‘no case’). As the comparisons are performed within individuals and within the same time window, this design controls for time-trends, time-invariant characteristics, and inter-patient variations, such as sex, age, and comorbidity. In this study, the calendar month was selected as a fixed time window and one day as the time unit. In the present study, a conditional quasi-Poisson regression model was applied. It has been shown that such a model is a flexible alternative to the conditional logistic model realization of the case-crossover method [20,21]. The advantage of using quasi-Poisson regression is that it controls for over dispersion and autocorrelation to fit the time-stratified case-crossover design. A mathematical representation of the model has the following form

(1) $$H_{i,s}|H_{.,s}\sim Multinomial\left(\pi_{i}=\frac{exp\left\{\beta^{T}x_{i}\right\}}{\sum_{j\in s}exp\left\{\beta^{T}x_{j}\right\}}\right),$$

where $H_{i,s}$ is the number of health events (counts) on the cluster and $H_{.,s}=\underset{i}{\sum }H_{i,s}$ is the sum of events calculated in each considered cluster. The notation in the formula is used as follows: event day *i*, *j* is an index on *S*, *β* is a row vector of coefficients, and *T* denotes transposition. A vector *x* contains variables of interest (air pollutants and weather factors). The parameters associated with clusters are eliminated by conditioning on the sum of events on each constructed cluster [21].

In the study, the health data were organized as daily counts of the number of ED visits for the considered adverse health conditions. The applied statistical method was realized using the *gnm* procedure (an R Package for Generalized Nonlinear Models; [22]). The model has the following transcription form

FitModel = *gnm* (EDCounts~AirPollutant + ns (RelativeHumidity,3) + ns (Temperature,3)).

Additionally, in the presented model, ns is the natural spline realized with 3 degrees of freedom. In the calculations the following options were included in the specification of the *gnm* routine: family = *quasipoisson*, eliminate = *factor* (Cluster). The variable EDCounts represents daily counts for the considering health data and cluster. Quasi-Poisson is used to model an overdispersed count variable. The realized approach was developed in the study conducted by Armstrong and colleagues [21] as a flexible alternative to the case-crossover method [18]. Conditional Poisson regression is realized on the clusters determined by a hierarchical structure of the calendar. The data were grouped by year, month, and day of week. The clusters group the same weekdays, thus one cluster includes 4 or 5 days. It is a technique very similar to the time-stratified case-crossover approach to organize case days and control days [19,20].

The strata (sub-groups of the patients) were defined by sex, age group (under 11, between 11 and 60, over 60 years of age), and season (warm and cold period). The results from all models are presented in the Supplementary Materials. A *p*-value of lower than 0.05 was considered statistically significant. The model results were also compared using a *p*-value < 0.001.

## 3. Results

The top frequencies of the number of ED visits by the considered chapters were as follows: 96,198 (28.9% of all in the corresponding chapter) for D12 (“Benign neoplasm of colon, rectum, anus and anal canal”), 36,305 (48.4%) for D64 (“Other anaemias”), 27,299 (25.6%) for E11 (“Non-insulin-dependent diabetes mellitus”), 512,573 (22.0%) for R10 (“Abdominal and pelvic pain”), respectively. Table 1 represent the top 20 frequencies in the four considered Chapters II–IV and XVIII, respectively.

The table (Table 1) demonstrates that over 50% of all ED visits in the corresponding chapters is achieved by the first 5, 2, 3, and 5 of their top frequencies. This indicates a measure of the variability in health problems classified in the chapters. The analyzed daily counts amalgamate all the ED visits that occurred in one day. For example, the results show that in the case of Chapter III (Endocrine, nutritional and metabolic diseases; ICD10: D50-D89) the following two health problems: “Other anaemias” and ”Sickle-cell disorders” are dominant among those considered in this chapter. As a consequence, they are most likely to participate in the daily counts. It is expected that associations will be detected even for counts that group various health conditions. The associations can be statistically significantly positive or negative, and neutral (no associations).

The majority of the results are provided in the Supplementary Materials. A series of tables, such as Table C00D48, Table D50D89, Table E00E90, and Table R00R99 represent the statistics on the number of daily ED visits over 4292 days of the study period. The tables illustrate the total number of ED visits, and their minimum, maximum, mean, median and two percentiles (25th and 75th) of daily frequencies. These characteristics are derived from 18 constructed strata and are presented in the tables. The following number of ED visits were identified by the considered four chapters: Neoplasms (Chapter II) 333,485 (female 176,234), Diseases of the blood and blood-forming organs and certain disorders involving the immune mechanism (Chapter III) 74,998 (41,850), Endocrine, nutritional and metabolic diseases (Chapter IV); 116,583 (61,282), and Symptoms, signs and abnormal clinical and laboratory findings, not elsewhere classified 2,325,328 (1,282,317). The counts related to the number of ED visits classified by ICD-10 codes R00–R99 are much higher than counts identified for other three chapters. Chapter XVIII covers a large spectrum of health conditions. For example, one subgroup (ICD-10 codes: R00–R09—“Symptoms and signs involving the circulatory and respiratory systems”) contains a very large spectrum of health conditions (R00—Abnormalities of heart beat, R01—Cardiac murmurs and other cardiac sounds, R02—Gangrene, not elsewhere classified, R03—Abnormal blood-pressure reading, without diagnosis, R04—Haemorrhage from respiratory passages, R05—Cough, R06—Abnormalities of breathing, R07—Pain in throat and chest, R09—Other symptoms and signs involving the circulatory and respiratory systems). In this case, the subgroup (ICD-10: R00–R09) has health conditions related to the circulatory and respiratory systems. This wide spectrum of diseases justifies the large number of ED visits in Chapter XVIII. These tables are presented in the Supplementary Materials at the following website https://github.com/szyszkowiczm/ICD10-C00D48-D50D89-E00E90-R00R99TORONTO, (accessed on 3 May 2022). The corresponding files also contain figures, which are maps of the associations for 2160 models.

The main goal of this work was to identify any possible associations between exposure to ambient air pollution and adverse health outcomes. The number of positive statistically significant associations obtained for the health problems is as follows: 260 (96), 285 (52), 198 (29), and 362 (97) for Chapters II, III, IV, and XVIII, respectively. The numbers were obtained for a *p*-value <0.05. The numbers in the parentheses are shown for a *p*-value <0.001.

**Figure 1:**
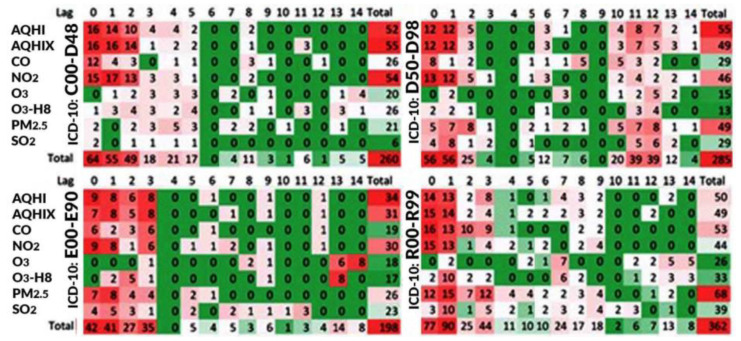
Frequencies of the numbers of positive statistically significant (*p*-value < 0.05) associations summarized by all strata (as per patients’ sex and age, and seasons). Chapters II–IV and XVIII (ICD-10 codes: C00–D48, D50–D89, E00–E90, and R00–R99) are based on the Toronto patients attending ED in a period between 2004 and 2015.

**Figure 2:**
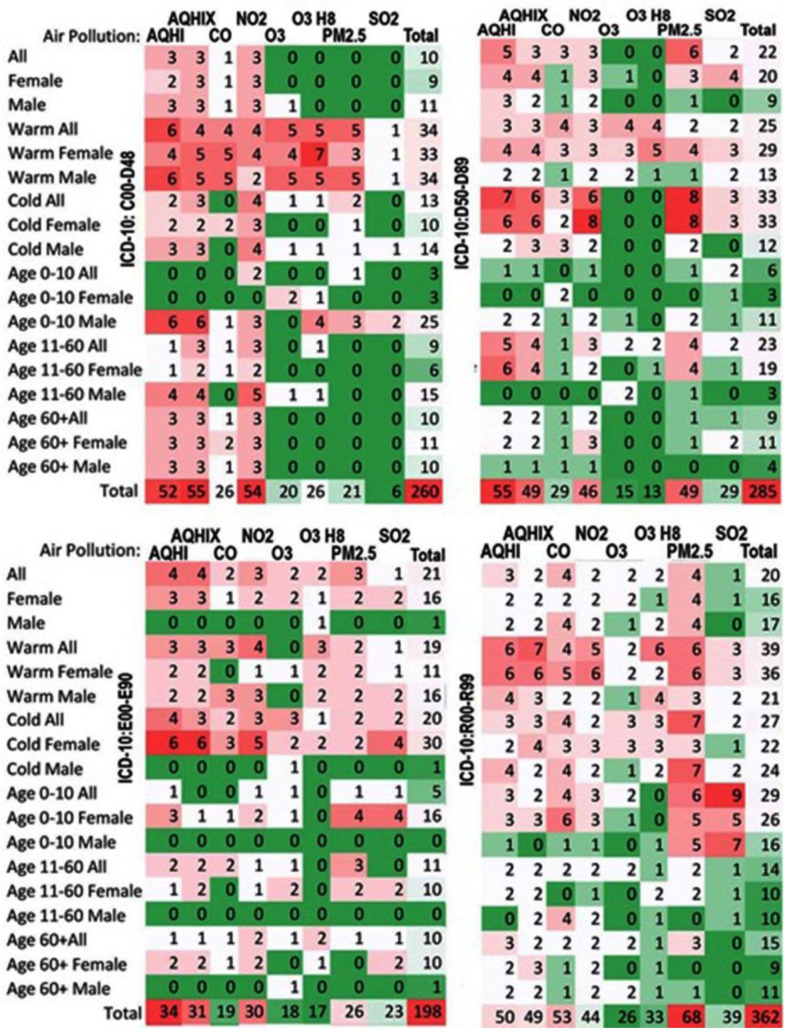
Frequencies of the numbers of positive statistically significant (*p*-value < 0.05) associations summarized by all-time lags expressed as days (0–14). Chapters II–IV and XVIII (ICD-10 codes: C00–D48, D50–D89, E00–E90, and R00–R99) are based on the Toronto patients attending ED in a period between 2004 and 2015.

**Figure 3:**
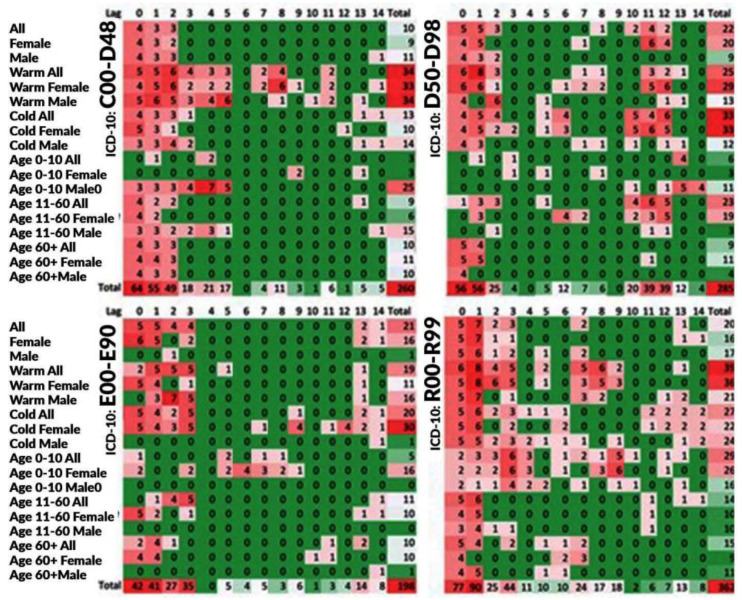
Frequencies of the numbers of positive statistically significant (*p*-value < 0.05) associations summarized by all air pollutants. Chapters II–IV and XVIII (ICD-10 codes: C00–D48, D50–D89, E00–E90, and R00–R99) are based on the Toronto patients attending ED in the period between 2004 and 2015.

Figure 1, Figure 2 and Figure 3 illustrate a summary of the frequency of positive statistically significant (*p*-value<0.05) associations. Each statistical model was constructed for three specifications, which were as follows: individual air pollutant, composite air health effects index, time lag, and strata expressed by patients’ sex and age and seasons. Thus, the results are constructed in the 3D space with these three coordinates. To identify the potential patterns of the associations, a kind of “summary” was created.

Figure 1 illustrates the results “summarized” by the strata. Thus, it shows the projection of 3D space onto a 2D plane with the axis air pollutant and their lags. In this situation, classification by strata is ignored (associations are summarized by strata). The cells show how many associations were obtained for the considered strata. The maximum value in a cell can be 18, in such a scenario, each stratum has an association. The figure shows that the indexes (AQHI, AQHIX) have high number of associations for health conditions classified in Chapters II–IV. The indexes represent multipollutant exposures. Their individual components have high associations with ED visits classified in Chapter II and IV for NO_2_ and for PM_2.5_ in Chapter III. For ED visits classified in Chapter XVII, fine particulate matter had the highest number of positive associations.

Figure 2 displays the same idea of the results in a 3D space projected onto a 2D plane. It shows the summaries for all considered time lags expressed as day, and thus, the maximum value in the cells can be 15 in a case if the associations were positive for all lags (0–14). The figure identifies stratum and air pollutants with the number of the positive statistically significant associations.

Figure 3 also presents the results on the plane with the axis stratum and lag. It summarizes the positive scores by all of the studied ambient air pollutants. The maximum value in the cells can be 8. This maximum value was obtained for lag 1 for two Chapters (III and XVIII; ICD-10 codes: D50–D89, R00–R99).

The applied method to illustrate the results from 2160 statistical models allows us to qualify the various associations. Similar values were grouped by colour. The figures indicate the patterns for the four analyzed ICD-10 Chapters. The statistics on the environmental data (air pollutants, temperature, and relative humidity) are presented in Table 2 (publication [23]) by two seasons and for the whole period.

Table 2, Table 3, Table 4 and Table 5 contain estimated relative risks (RR) and their 95% confidence intervals (CI) reported for an increase in concentration by a one interquartile range (IQR). The presented values were chosen for the studied ambient air pollutants and time lags, which have the highest scores of the positive associations. They are the AQHIX, AQHI, and PM_2.5_ for lags 0, 1, and 2.

The tables provide estimations of RRs and 95CIs. The values with a low boundary of the confidence interval of greater than 1 are positive statistically significant. As in the total, there are 4 × 2160 models the idea of the color maps demonstrates this well.

The results obtained from all models (2160) are given at the following website (https://github.com/szyszkowiczm/ICD10-C00D48-D50D89-E00E90-R00R99TORONTO, (accessed on 3 May 2022)). The numerical results are accompanied by maps with values 0, 1, and −1. These values qualify the associations as none and statistically significantly positive and negative, respectively. The maps are figures, which also show these associations in color (0/white, 1/red, and −1/green). The corresponding files are: C00D48Map.jpg, C00D48TorontoRRisk.csv; D50D89Map.jpg, D50D89TorontoRRisk.csv; E00E90Map.jpg, E00E90TorontoRRisk.csv; R00R99Map.jpg, and R00R99TorontoRRisk.csv as found at the abovementioned website. In addition, as was already mentioned above, Table C00D48, Table D50D89, Table E00E90, and Table R00R99, stored as pdf files, present tables with the top 20 frequencies and the corresponding maps of the associations. Four additional files HistTempRHum.jpg, HistAQHI-AQHIX-CO-NO2.jpg, HistO3-O3H8-PM25-SO2.jpg, and TorontoMapStation.jpg illustrate histograms of the used environmental factors and a map of Toronto.

## 4. Discussion

Numerous studies have shown positive correlations between short-term changes in ambient air pollution concentrations and cardiopulmonary health outcomes. The number of related publications very well represent the situation in the thematic and cause-specific-oriented studies [1]. The ratio of these numbers, compared as pulmonary- versus cardiac-related publications, strongly indicate a traditional approach in environmental epidemiology. It is a common-sense assumption that the “inhalation of a polluted air affects the human respiratory system”. A recent search for the corresponding publications in PubMed with the keywords “air pollution pulmonary” resulted in over 12,000 results. A similar search with the string “air pollution cardiac” showed 3838 results. In contrast, a search with the words “air pollution depression“ returned 593 results, and the string “air pollution mental” gives 977 results in total. These numbers very well characterize the subject and focus of the studies in the environmental domain.

The presented work considers four different large groups of health conditions organized in the separated Chapters of the ICD-10 classifications. These health outcomes are different but in the present study were “exposed” to the same environmental data. In addition, the same statistical methodology was applied to investigate the associations. This approach can elucidate and compare potential effects. The intention here is to show that other diseases are also associated with exposure to ambient air quality. The results demonstrate very consistent associations for time lags from 0 to 3 or 4 days for the considered health condition categories.

The results present summaries of the relations in the form of color maps. They help to visually identify the patterns of the associations. The tabulated relative risks and their 95% confidence intervals provide quantitative estimations of the risks. There are few publications in environmental epidemiology related to the health conditions analyzed here. As a consequence, it is difficult to compare the presented results with the results reported by other studies. Here, the burden of diseases in relation to urban ambient air pollution concentrations is mainly presented.

There are a few noteworthy advantages of the present study. The data used are very reliable. The health data were collected and administrated by CIHI, which are very well organized and classified by professional nosologists [13]. Environmental data, air pollutant measurements and weather factors, were measured by the site-fixed monitoring stations network spread around Toronto [15]. The statistical methodology used, in its kernel as conditional Poisson regression, is very reliable and relatively fast.

The main limitation of this study is that it does not control for individual exposure, its duration and air pollutant concentrations. It assumed the same exposure among patients attending ED for all health outcomes cases. The study also does not adjust for heterogeneity of exposure to air pollution in a population. Individuals are affected by numerous sources of pollution and usually it is not registered by an ambient air pollution monitor. Such exposure might be due to smoking habits, second-hand smoke exposure, cooking emissions, wildfires, occupational exposure, and proximity to major roads. In the study, the multiplicity of comparisons performed (2160 models) may introduce a possibility of erroneous associations. The presented figures in the form of color maps show a consistent pattern of the associations for four ICD-10 Chapters. The strongest effects are present for the exposures lagged by 0 or 1 day.

## 5. Conclusions

In the present study, we took as an implicit assumption that daily cases are mixtures of health events which have associations with air pollution and those that do not have. A very low resolution is here applied with respect to health conditions. A consideration of specific health problem should increase the number of associations, as is the case when analyzing all respiratory vs. asthma and ozone concentration. As demonstrated in the Figures, response to exposure is present and many cases may play the role of white noise.

The present study suggests that acute changes in urban air pollutant concentrations may affect the numbers of the ED visits for the diseases classified into four different categories. The results demonstrated that air pollution concentrations have the strongest correlations for exposures that are lagged from 0 to 4 days. The study can be considered as an n=an initial exploration of the specific health conditions from the used Chapters. The direction suggested here is from general to specific health conditions rather than in the opposite direction, such as from asthma to respiratory conditions.

The results of the study also encourage a similar study. Traditionally, the majority of the studies in environmental epidemiology have focused on cardiopulmonary health conditions. Here, it has been shown that other health problems are also related to ambient air pollution concentrations.

## Figures and Tables

**Table 1:** Top 20 frequencies of ED visits classified in Chapters II and III.

**C00D48**	**Frequency**	**%**	**D50D89**	**Frequency**	**%**
D12	96,198	28.9	D64	36,305	48.4
C44	26,088	7.8	D57	10,261	13.7
C50	18,970	5.7	D50	8490	11.3
C67	16,003	4.8	D70	6390	8.5
D25	13,043	3.9	D69	4350	5.8
C34	12,117	3.6	D68	3152	4.2
D41	11,075	3.3	D61	1473	2.0
C18	8324	2.5	D86	659	0.9
D17	8269	2.5	D72	589	0.8
C61	7776	2.3	D75	500	0.7
D24	7413	2.2	D53	387	0.5
D22	7259	2.2	D66	367	0.5
C78	5382	1.6	D56	358	0.5
D05	4703	1.4	D58	312	0.4
C79	4654	1.4	D59	292	0.4
D37	4462	1.3	D73	217	0.3
D06	4310	1.3	D52	194	0.3
D23	3964	1.2	D62	142	0.2
C20	3855	1.2	D51	123	0.2
D27	3798	1.1	D80	94	0.1
**E00E90**	**Frequency**	**%**	**R00R99**	**Frequency**	**%**
E11	27,299	25.6	R10	512,573	22.0
E87	19,190	18.0	R07	398,248	17.1
E86	13,929	13.0	R51	111,339	4.8
E14	13,686	12.8	R50	111,089	4.8
E10	13,302	12.5	R42	99,923	4.3
E16	4524	4.2	R06	94,296	4.1
E04	3926	3.7	R11	92,029	4.0
E83	3827	3.6	R55	85,902	3.7
E05	1105	1.0	R31	75,426	3.2
E03	775	0.7	R00	55,931	2.4
E61	539	0.5	R53	55,390	2.4
E06	494	0.5	R56	52,139	2.2
E27	430	0.4	R04	51,807	2.2
E88	372	0.4	R05	50,436	2.2
E28	294	0.3	R33	44,464	1.9
E85	296	0.3	R21	42,862	1.8
E65	275	0.3	R45	36,870	1.6
E07	271	0.3	R22	31,312	1.4
E84	250	0.2	R20	28,711	1.2
E80	228	0.2	R41	26,015	1.1

**Table 2:** Relative risks (RR) and their 95% confidence intervals (95%CI) for an increase in the AQHI-X level by a one interquartile range (IQR = 1.5). ICD-10 codes: C00–D48; Chapter II Neoplasms Data recorded in Toronto, Canada for the period between April 2004 and December 2015.

Lags	Lag 0		Lag 1		Lag 2	
Strata	RR	95%CI	RR	95%CI	RR	95%CI
All	1.048	(1.032, 1.063)	1.032	(1.016, 1.047)	1.022	(1.006, 1.037)
Female	1.050	(1.034, 1.067)	1.031	(1.014, 1.047)	1.021	(1.004, 1.038)
Male	1.045	(1.028, 1.062)	1.033	(1.016, 1.050)	1.022	(1.005, 1.040)
Warm All	1.031	(1.019, 1.044)	1.020	(1.008, 1.033)	1.025	(1.012, 1.038)
Warm Female	1.031	(1.017, 1.045)	1.020	(1.006, 1.034)	1.027	(1.013, 1.042)
Warm Male	1.032	(1.018, 1.046)	1.021	(1.007, 1.035)	1.023	(1.008, 1.037)
Cold All	1.070	(1.052, 1.090)	1.048	(1.029, 1.067)	1.021	(1.003, 1.040)
Cold Female	1.078	(1.058, 1.098)	1.046	(1.027, 1.066)	1.017	(0.998, 1.037)
Cold Male	1.063	(1.042, 1.083)	1.049	(1.029, 1.070)	1.025	(1.005, 1.045)
Age 0–10 All	1.023	(0.958, 1.092)	1.063	(0.996, 1.134)	1.059	(0.991, 1.133)
Age 0–10 Female	0.955	(0.879, 1.037)	1.025	(0.943, 1.115)	1.020	(0.937, 1.110)
Age 0–10 Male0	1.094	(1.010, 1.184)	1.099	(1.017, 1.188)	1.099	(1.013, 1.192)
Age 11–60 All	1.050	(1.033, 1.067)	1.025	(1.008, 1.042)	1.019	(1.002, 1.036)
Age 11–60 Female	1.047	(1.028, 1.066)	1.020	(1.002, 1.039)	1.015	(0.996, 1.034)
Age 11–60 Male	1.055	(1.034, 1.076)	1.032	(1.011, 1.054)	1.025	(1.004, 1.047)
Age 60+ All	1.046	(1.030, 1.063)	1.037	(1.020, 1.053)	1.023	(1.007, 1.040)
Age 60+ Female	1.055	(1.036, 1.074)	1.042	(1.023, 1.061)	1.027	(1.008, 1.046)
Age 60+Male	1.040	(1.022, 1.058)	1.032	(1.014, 1.051)	1.020	(1.002, 1.038)

**Table 3:** Estimated relative risks (RR) and their 95% confidence intervals (95%CI) for an increase in the AQHI level by a one interquartile range (IQR = 1.0). ICD-10 codes: D50–D89; Chapter III Diseases of the blood and blood-forming organs and certain disorders involving the immune mechanism. Data recorded in Toronto, Canada for the period between April 2004 and December 2015.

Lags	Lag 0		Lag 1		Lag 2	
Strata	RR	95%CI	RR	95%CI	RR	95%CI
All	1.025	(1.011, 1.039)	1.026	(1.012, 1.040)	1.014	(1.000, 1.028)
Female	1.024	(1.007, 1.042)	1.030	(1.012, 1.048)	1.007	(0.989, 1.025)
Male	1.026	(1.006, 1.046)	1.021	(1.001, 1.042)	1.023	(1.003, 1.043)
Warm All	1.025	(1.012, 1.038)	1.022	(1.009, 1.035)	1.013	(1.000, 1.026)
Warm Female	1.029	(1.012, 1.046)	1.027	(1.010, 1.045)	0.999	(0.982, 1.016)
Warm Male	1.021	(1.002, 1.039)	1.015	(0.997, 1.034)	1.031	(1.012, 1.050)
Cold All	1.027	(1.012, 1.042)	1.032	(1.017, 1.047)	1.017	(1.002, 1.032)
Cold Female	1.022	(1.004, 1.042)	1.035	(1.015, 1.054)	1.018	(0.999, 1.037)
Cold Male	1.034	(1.012, 1.056)	1.028	(1.006, 1.050)	1.015	(0.993, 1.037)
Age 0–10 All	1.037	(0.983, 1.094)	1.032	(0.978, 1.089)	1.001	(0.948, 1.057)
Age 0–10 Female	1.036	(0.964, 1.115)	1.015	(0.943, 1.093)	0.953	(0.885, 1.027)
Age 0–10 Male0	1.039	(0.969, 1.115)	1.048	(0.977, 1.124)	1.042	(0.970, 1.119)
Age 11–60 All	1.014	(0.994, 1.034)	1.026	(1.005, 1.046)	1.024	(1.004, 1.045)
Age 11–60 Female	1.017	(0.991, 1.043)	1.034	(1.008, 1.061)	1.022	(0.996, 1.049)
Age 11–60 Male	1.010	(0.979, 1.041)	1.014	(0.983, 1.046)	1.028	(0.997, 1.060)
Age 60+ All	1.033	(1.014, 1.052)	1.026	(1.007, 1.045)	1.007	(0.988, 1.026)
Age 60+ Female	1.030	(1.005, 1.055)	1.028	(1.003, 1.053)	0.999	(0.975, 1.024)
Age 60+Male	1.036	(1.009, 1.063)	1.024	(0.997, 1.052)	1.017	(0.990, 1.044)

**Table 4:** Relative risks (RR) and their 95% confidence intervals (95%CI) for an increase in the AQHI level by a one interquartile range (IQR = 1.0). ICD-10 codes: E00–E90; Chapter IV Endocrine, nutritional and metabolic diseases. Data recorded in Toronto, Canada for the period between April 2004 and December 2015.

Lags	Lag 0		Lag 1		Lag 2	
Strata	RR	95%CI	RR	95%CI	RR	95%CI
All	1.014	(1.003, 1.025)	1.013	(1.003, 1.024)	1.011	(1.001, 1.022)
Female	1.024	(1.009, 1.039)	1.018	(1.004, 1.033)	1.009	(0.995, 1.024)
Male	1.004	(0.989, 1.019)	1.008	(0.993, 1.023)	1.014	(0.999, 1.029)
Warm All	1.007	(0.997, 1.018)	1.013	(1.003, 1.023)	1.012	(1.002, 1.022)
Warm Female	1.019	(1.006, 1.033)	1.016	(1.002, 1.030)	1.003	(0.989, 1.016)
Warm Male	0.995	(0.981, 1.009)	1.009	(0.995, 1.024)	1.022	(1.008, 1.037)
Cold All	1.023	(1.011, 1.035)	1.012	(1.001, 1.024)	1.012	(1.000, 1.024)
Cold Female	1.030	(1.014, 1.047)	1.020	(1.003, 1.036)	1.021	(1.004, 1.037)
Cold Male	1.015	(0.999, 1.031)	1.005	(0.989, 1.021)	1.003	(0.987, 1.019)
Age 0–10 All	1.065	(1.001, 1.133)	1.026	(0.963, 1.092)	1.010	(0.948, 1.075)
Age 0–10 Female	1.098	(1.016, 1.187)	0.988	(0.912, 1.072)	0.974	(0.898, 1.057)
Age 0–10 Male0	1.034	(0.955, 1.120)	1.061	(0.980, 1.147)	1.040	(0.960, 1.127)
Age 11–60 All	1.014	(0.998, 1.030)	1.010	(0.994, 1.026)	1.018	(1.002, 1.035)
Age 11–60 Female	1.028	(1.005, 1.051)	1.016	(0.994, 1.039)	1.021	(0.998, 1.045)
Age 11–60 Male	1.001	(0.979, 1.023)	1.004	(0.982, 1.026)	1.016	(0.993, 1.038)
Age 60+ All	1.012	(0.998, 1.027)	1.015	(1.001, 1.030)	1.006	(0.992, 1.021)
Age 60+ Female	1.019	(1.000, 1.038)	1.021	(1.002, 1.040)	1.003	(0.984, 1.022)
Age 60+Male	1.005	(0.984, 1.025)	1.009	(0.988, 1.030)	1.011	(0.990, 1.032)

**Table 5:** Relative risks (RR) and their 95% confidence intervals (CI) for an increase in fine particulate matter (PM_2.5_) level by a one interquartile range (IQR = 6.5 ppb). ICD-10 codes: R00–R99; Chapter XVIII Symptoms, signs and abnormal clinical and laboratory findings, not elsewhere classified. Data recorded in Toronto, Canada for the period between April 2004 and December 2015.

Lags	Lag 0		Lag 1		Lag 2	
Strata	RR	95%CI	RR	95%CI	RR	95%CI
All	1.005	(1.002, 1.008)	1.006	(1.003, 1.009)	1.004	(1.001, 1.007)
Female	1.005	(1.002, 1.009)	1.006	(1.002, 1.009)	1.004	(1.001, 1.008)
Male	1.004	(1.001, 1.008)	1.006	(1.002, 1.009)	1.003	(0.999, 1.007)
Warm All	1.002	(0.999, 1.005)	1.005	(1.002, 1.008)	1.002	(0.999, 1.005)
Warm Female	1.002	(0.999, 1.006)	1.005	(1.002, 1.009)	1.004	(1.001, 1.008)
Warm Male	1.001	(0.997, 1.004)	1.004	(1.001, 1.008)	1.000	(0.996, 1.003)
Cold All	1.007	(1.004, 1.010)	1.007	(1.004, 1.009)	1.004	(1.001, 1.007)
Cold Female	1.008	(1.004, 1.011)	1.006	(1.003, 1.010)	1.004	(1.000, 1.007)
Cold Male	1.006	(1.003, 1.010)	1.007	(1.003, 1.010)	1.005	(1.002, 1.009)
Age 0–10 All	1.013	(1.005, 1.022)	1.011	(1.003, 1.020)	1.011	(1.002, 1.019)
Age 0–10 Female	1.011	(1.000, 1.023)	1.009	(0.998, 1.020)	1.011	(1.000, 1.022)
Age 0–10 Male0	1.015	(1.005, 1.025)	1.013	(1.003, 1.023)	1.010	(1.000, 1.021)
Age 11–60 All	1.004	(1.001, 1.007)	1.005	(1.002, 1.009)	1.003	(1.000, 1.006)
Age 11–60 Female	1.006	(1.002, 1.010)	1.006	(1.002, 1.010)	1.003	(0.999, 1.007)
Age 11–60 Male	1.002	(0.998, 1.006)	1.004	(1.000, 1.008)	1.003	(0.998, 1.007)
Age 60+ All	1.004	(1.000, 1.008)	1.006	(1.001, 1.010)	1.003	(0.998, 1.007)
Age 60+ Female	1.004	(0.999, 1.009)	1.004	(0.999, 1.010)	1.004	(0.999, 1.009)
Age 60+Male	1.005	(0.999, 1.010)	1.007	(1.001, 1.012)	1.001	(0.996, 1.007)

## Data Availability

Air pollution data: National Air Pollution Surveillance Program. https://www.canada.ca/en/environment-climate-change/services/air-pollution/monitoring-networks-data/national-air-pollution-program.html, (accessed on 10 March 2022). Health data: https://www.cihi.ca/en/national-ambulatory-care-reporting-system-metadata, (accessed on 10 March 2022).

## Supplementary Materials

The supporting information can be downloaded at the following website: https://github.com/szyszkowiczm/ICD10-C00D48-D50D89-E00E90-R00R99TORONTO (accessed on 10 March 2022).
